# Comprehensive analysis of mycobacterium tuberculosis antigen-specific CD4^+^ T cell responses restricted by single HLA class II allotype in an individual

**DOI:** 10.3389/fimmu.2022.897781

**Published:** 2022-07-28

**Authors:** Yong-Hun Lee, You-Seok Hyun, Hyeong-A Jo, In-Cheol Baek, Sun-Mi Kim, Hyun-Jung Sohn, Tai-Gyu Kim

**Affiliations:** ^1^ Department of Microbiology, College of Medicine, The Catholic University of Korea, Seoul, South Korea; ^2^ Department of Biomedicine and Health Sciences, College of Medicine, The Catholic University of Korea, Seoul, South Korea; ^3^ Catholic Hematopoietic Stem Cell Bank, College of Medicine, The Catholic University of Korea, Seoul, South Korea

**Keywords:** *Mycobacterium tuberculosis*, HLA class II allotype, CD4^+^ T cell, allotype dominance, intra-individual dominance, *ex vivo* ELISPOT, cultured ELISPOT

## Abstract

*Mycobacterium tuberculosis* infection is generally asymptomatic as latent tuberculosis, but it is still known as the world’s leading bacterial cause of death. The diagnosis of latent tuberculosis infection relies on the evidence of cellular immunity to mycobacterial antigens. Since the association between HLA class II and tuberculosis infection has been reported in several population groups, a detailed study on the CD4^+^ T cell response to major tuberculosis antigens is needed. To elucidate which HLA class II allotypes in an individual are preferentially used in tuberculosis, CD4^+^ T cells specific to TB10.4, Ag85b, ESAT-6, and CFP-10 of *Mycobacterium tuberculosis* antigens were analyzed comprehensively. A total of 33 healthy donors were analyzed by *ex vivo* and cultured ELISPOT using panels of artificial antigen-presenting cells expressing a single HLA class II allotype. The CD4^+^ T cell responses were increased by an average of 39-fold in cultured ELISPOT compared with *ex vivo* ELISPOT. In *ex vivo* and cultured ELISPOT, CD4^+^ T cell responses showed significantly higher by HLA-DR than those of HLA-DQ and HLA-DP locus. In cultured ELISPOT, 9 HLA-DR allotypes, 4 HLA-DQ allotypes, and 3 HLA-DP allotypes showed positive CD4^+^ T cell responses. Among ten donors with positive CD4^+^ T cell responses when tested for mixed Mycobacterium tuberculosis antigens, seven donors were positive for only a single allotype, and three were positive for two allotypes in an individual. However, only one allotype was used for a single antigen-specific response when a single tuberculosis antigen was used individually. These results on the distribution of HLA class II allotypes showing high CD4^+^ T-cell responses to *Mycobacterium tuberculosis* antigens and the intra-individual allotype dominance will provide valuable information for understanding the immunobiology and immunogenetics of tuberculosis, which can contribute to the development of more effective vaccines.

## Introduction

Tuberculosis (TB) is a communicable disease caused by infection of *Mycobacterium tuberculosis (M. tuberculosis*). Most people who are latently infected with *M. tuberculosis* are clinically asymptomatic and estimated to make up a quarter of the global population (1). The 5–10% of people with latent TB develop active disease sometime during their lives. *M. tuberculosis*-specific CD4^+^ T cell response is crucial for controlling the replication of *M. tuberculosis* (2).

The human leukocyte antigen (HLA) class II molecules present antigens to CD4^+^ T cells and are preferentially expressed by professional antigen-presenting cells (APC), including dendritic cells, macrophages. There are three classical HLA class II molecules encoded by HLA-DR, -DQ, and -DP locus, which have high polymorphism. This polymorphism provides an advantage in the survival of a population against the pathogens recognized by T cells (3). HLA molecules present epitopes, which are peptides known to have immunodominance and are more frequently recognized than others. It suggests being related to peptide-binding repertoires of affinities, different sizes, and immunogenicities (4, 5). Association between HLA class II and TB has been reported in several population groups. Although the results have been inconsistent among various ethnic groups (6), the association of HLA-DR2 or DRB1*15 with TB has been found commonly in Indians (7–10), Indonesian (11), and Russia (12). DRB1*08:03 and DQB1*06:01 alleles have been reported to be associated with disease progression of TB in Korean (13).

The most common tests for diagnosis of latent TB infection are the intradermal tuberculin test and IFN-γ release assay, which relies on the evidence of cellular immune response to mycobacterial antigens. The intradermal tuberculin test is based on delayed-type hypersensitivity skin reactivation to the proteins from mycobacterial culture supernatants (14, 15). The IFN-γ release assay detects the IFN-γ produced by T cells stimulated with mycobacterial antigens, which include the early secretory antigenic target (ESAT-6) and the 10-kDa culture filtrate protein (CFP-10). In addition to these antigens, the extensively characterized immunogenic antigens were TB10.4 and Ag85b inducing a strong response against *M. tuberculosis* (16–21).

The effector and memory T cells specific to an antigen can be characterized by are *ex vivo* and cultured IFN-γ enzyme-linked immunosorbent spot-forming cell assay (ELISPOT) (22–24). The *ex vivo* ELISPOT is an approach for measuring effector T cell responses (25), and the cultured ELISPOT measures T cell precursors with a proliferative capacity (26). The memory T cell responses detected by cultured ELISPOT correlate with the protection against TB (27, 28), malaria (24, 29, 30), hepatitis B virus (31), and HIV (26).

We investigated the CD8^+^ and CD4^+^ T cell responses to the cytomegalovirus (CMV)-pp65 antigen by a single HLA allotype in each individual previously. The study elucidated the intra-individual dominance that only a limited number of HLA allotypes is used for T cell responses in an individual (32, 33). Here, the responses of CD4^+^ T cells to four antigens of *M. tuberculosis* in healthy donors were analyzed comprehensively using the panels of artificial APCs (aAPC) expressing a single HLA class II allotype. A few HLA class II allotypes were used preferentially in CD4^+^ T cell responses specific to TB. For comparison of effector and memory CD4^+^ T cell response, CD4^+^ T cell responses were measured by *ex vivo* ELISPOT and cultured ELISPOT. The data elucidated the distribution of HLA class II allotypes with strong responses to M. tuberculosis antigens. Moreover, the CD4^+^ T cells responded to one allotype, which mainly responded to one of *M. tuberculosis* antigens in an individual.

## Materials and methods

### Human blood samples

This study was approved by Institutional Review Board of the Catholic University of Korea (MC21SESI0005). All healthy donors provided written informed consent before participation in this study. Healthy donors who did not have chronic diseases such as diabetes, thyroid, and hypertension and were not taking medications. A complete blood cell count test and additional tests for HBV, HCV, HIV-1, HIV-2, and syphilis were performed on all donors. The absence of active tuberculosis was confirmed by regular medical check-up. The participants are ranged from 26.4 ± 4.0 years of age and composed of 6 females and 27 males. PBMCs were isolated by density gradient centrifugation using Ficoll–Hypaque (GE Healthcare). CD4^+^ T cells were isolated and CD8^+^ T cells were depleted using magnetic microbeads (AutoMacs Pro separator; Miltenyi Biotec), and the purity of the CD4^+^ T cells and CD8^-^ PBMCs was confirmed with flow cytometry (CD8^+^ T cells in CD8^-^ PBMC: 0.7% ± 0.7%, **Supplementary Figure S1**). CD8^+^ T cells were depleted to precisely measure the response by CD4^+^ T cells. Suspended CD4^+^ T cells and CD8^-^ PBMCs in fetal bovine serum (Gibco) containing 10% dimethyl sulfoxide (Mylan) and 50% RPMI 1640 medium (Lonza) were cryopreserved in liquid nitrogen until ELISPOT assay or cultured. The HLA was then genotyped using sequencing-based typing and next-generation sequencing at the Catholic Hematopoietic Stem Cell Bank (**Table 1**) (NCBI BioProject Accession: PRJNA721949) (MiSeqDx, Illumina), as previously described (32, 33).

**Table 1:** Genotypes of HLA class II in 33 healthy donors.

Donor	Sex	Age	DRB1*	DRB1*	DQA1*/DQB1*	DQA1*/DQB1*	DPA1*/DPB1*	DPA1*/DPB1*
HD01	M	24	08:03	12:01	01:03/06:01		01:03/04:01	01:03/03:01
HD02	F	31	01:01	15:02	01:01/05:01	01:03/06:01	01:03/04:02	01:03/03:01
HD03	M	31	08:02		01:03/06:03		01:03/02:01	02:02/05:01
HD04	M	31	04:03	13:02	03:01/03:02	01:02/06:04	02:02/05:01	01:03/04:01
HD05	M	26	13:02	16:02	01:02/06:09		01:03/02:02	02:02/05:01
HD06	M	32	04:04	09:01	03:02/03:03		01:03/04:02	02:02/05:01
HD07	M	31	13:02	15:01	01:02/06:02	01:02/06:09	02:02/05:01	02:02/03:01
HD08	F	31	03:01	12:02	05:01/02:01	06:01/03:01	01:03/03:01	02:02/05:01
HD09	M	18	08:02	14:54	01:04/05:02	03:01/03:02	01:03/04:02	02:02/05:01
HD10	M	31	04:06	09:01	03:01/03:02	03:02/03:03	02:02/05:01	
HD11	M	25	07:01	11:01	02:01/02:02	05:05/03:01	02:01/13:01	02:02/02:01
HD12	M	26	09:01	12:01	03:02/03:03		01:03/02:01	02:02/05:01
HD13	M	21	03:01	04:03	03:01/03:02	05:01/02:01	01:03/04:01	02:02/05:01
HD14	M	27	07:01	15:01	01:02/06:02	02:01/02:02	01:03/04:02	02:01/13:01
HD15	M	28	08:03	15:01	01:02/06:02	01:03/06:01	01:03/04:02	02:02/05:01
HD16	M	21	08:03	09:01	01:03/06:01	03:02/03:03	02:02/05:01	
HD17	M	22	12:01	15:01	01:02/06:02		02:02/05:01	
HD18	M	21	04:03	14:05	01:04/05:03	03:01/03:02	01:03/02:01	02:01/05:01
HD19	M	28	01:01	12:01	01:01/05:01	05:05/03:01	02:02/05:01	
HD20	F	23	03:01	08:03	05:01/02:01	06:01/03:01	01:03/02:01	01:03/04:01
HD21	M	22	14:05	15:01	01:02/06:02	01:04/05:03	01:03/02:01	02:02/05:01
HD22	M	29	04:06	08:03	03:01/03:02	06:01/03:01	01:03/02:01	02:02/05:01
HD23	M	22	08:02	14:06	03:01/03:02	05:03/03:01	02:01/13:01	02:02/05:01
HD24	F	31	04:05	07:01	02:01/02:02	03:03/04:01	01:03/02:01	02:02/05:01
HD25	F	29	07:01		01:04/05:02	02:01/02:02	01:03/02:01	02:02/05:01
HD26	M	30	12:01	15:01	01:02/06:02		01:03/02:01	
HD27	F	30	12:01		03:01/03:02	01:01/05:01	02:02/05:01	02:01/09:01
HD28	M	25	04:06	14:05	01:04/05:03	03:01/03:02	01:03/02:01	02:02/02:01
HD29	M	25	04:05	08:03	01:03/06:01	03:03/04:01	01:03/02:01	02:02/02:02
HD30	M	28	13:02	14:54	01:02/06:04	01:04/05:02	01:03/02:01	01:03/04:01
HD31	M	21	08:03	12:02	06:01/03:01		01:03/02:01	02:02/02:01
HD32	M	29	04:05	07:01	02:01/02:02	03:03/04:01	02:01/17:01	02:02/05:01
HD33	M	23	09:01	13:02	01:02/06:04	03:02/03:03	01:03/04:01	02:02/05:01

*Notation of the HLA allele.

### The panels of aAPCs expressing a single HLA class II allotype

The panels of aAPCs expressing a single HLA class II allotype were established in the previous study (32, 33). Briefly, cDNAs of HLA-DRA allele, 21 HLA-DRB1 alleles, 10 HLADQA1 alleles, 11 HLA-DQB1 alleles, 3 HLA-DPA1 alleles and 11 HLA-DPB1 alleles were obtained from lymphoblastoid cell lines of donors (740902.50; Macherey Nagel, RT300M; Enzynomics). PCR products including cDNA sequences of HLA class II alleles were cloned into the pCDH lentivector (#CD523A-1; System Biosciences) using In-Fusion Cloning (EZ015TL; Enzynomics). The sequences were verified by Sanger sequencing. 293TN cells (System Biosciences) were transfected (Lipofectamine 2000; Invitrogen) with psPAX2 (RRID: Addgene_12260), pMD.2G (RRID: Addgene_12259), and pCDH encoding a single HLA class II allele. After 48h the supernatant was harvested. The K562-based aAPCs, which do not express HLA molecules, were transduced with lentiviruses encoding an alpha and a beta chain at a multiplicity of infection (MOI) of 20. The transduced aAPCs were isolated by fluorescence-activated cell sorting (FACSAria Fusion, BD Biosciences) using the following monoclonal antibodies; HLA-DR (clone G46-6; RRID: AB_1727527), HLA-DQ (clone Tü169; RRID: AB_2738963, ü39; RRID: AB_395940), and HLA-DP (clone B7/21).The aAPCs expressing a single HLA class II allotype were then cultured in RPMI 1640 supplemented with 2 mM L-glutamine, 100 U/ml penicillin–streptomycin–amphotericin B mixture (Lonza), and 10% fetal bovine serum (Gibco). The expression of HLA class II on aAPCs was confirmed using flow cytometry (FACSCanto, BD Biosciences). All HLA-DR alpha chain were omitted because DRA1*01:01 alone was used.

### Peptide and pulsing

5×10^4^ or 5×10^3^ aAPCs were loaded with 60 nM of 15 -mer amino acid spanning the entire *M. tuberculosis* TB10.4, Ag85b, ESAT-6 and CFP-10 protein with 11 -mer amino acid overlap (JPT Peptide Technologies) for 3h in serum-free medium in a 96-well plate. The peptide loaded aAPCs were washed three times with serum-free medium using centrifuge and microplate washer (405LSR; BioTek).

### *Ex vivo* IFN-g ELISPOT

Briefly, an ELISPOT plates (Human IFN-γ ELISPOT set, BD, USA) were coated with IFN-γ capture antibody and incubated overnight at 4°C. The plates were washed 3 times with phosphate-buffered saline (PBS) and then blocked with RPMI 1640. After 2h the blocking, the plates were washed with PBS. The 5×10^5^ CD4^+^ T cells were co-incubated with peptide-pulsed or not 5×10^4^ aAPCs for 20h. The cells were removed and the plates were washed 3 times with 0.05% Tween 20/PBS using microplate washer (405LSR; BioTek). Biotinylated detection antibody for IFN-γ was added and incubated for 2h at 37°C. After 4 times washes with 0.05% Tween 20/PBS, avidin-horseradish peroxidase was added and plates were incubated for 1h at 37°C. The plates were washed 4 times with 0.05% Tween 20/PBS, followed by 2 washes with PBS and the addition of AEC substrate (BD) per well. The reaction was stopped by washing with deionized water, and the plates were dried overnight. The spot forming units were counted using an AID ELISPOT Reader System (AID Diagnostika GmbH). The frequencies of an HLA allotype-restricted CD4^+^ T cell response to antigens were calculated as [(response to aAPCs expressing HLA pulsed with peptide pools) − (response to aAPCs expressing HLA)] − [(response to aAPCs pulsed with peptide pools) − (response to aAPCs)], as previously described (32, 33).

### Cultured ELISPOT

On Day 0, isolated CD4^+^ T cells and CD8^-^ PBMCs were mixed 2:1 (8-12×10^6^) at 4×10^6^/ml. Each *M. tuberculosis* antigen peptide pools 0.1ug/ml were added to RPMI 1640 supplemented with 2 mM L-glutamine, 100 U/ml Penicillin-Streptomycin-Amphotericin B Mixture (Lonza), 10% fetal bovine serum (Gibco), 500U/ml IFN-γ (LG Chemical) and 200U/ml hIL-2 (JW CreaGene) and incubated in 5% CO_2_, 37°C. On Day 1, 4, and 7, hIL-2 was added to medium 200U/ml. On Day 11, cells were sub-cultured to replenish the medium at 2×10^6^/ml with hIL-2 200U/ml. On Day 14, cells were harvested and measured IFN-γ secretion by ELISPOT assay. The 5×10^4^ cultured CD4^+^ T cells and 5×10^3^ antigen-pulsed or not aAPC were co-incubated for 20h at 37°C. *M. tuberculosis* antigens-specific responses of cultured CD4^+^ T cell responses were measured by IFN-γ ELISPOT assay as described in cultured ELISPOT assay. To compare *ex vivo* and cultured ELISPT, the frequencies of each ELISPOT were adjusted to account for differences in cell numbers and proliferation folds among the donors. It was calculated as (SFCs/5×10^4^ CD4^+^ T cells) × (1×10^6^) × proliferation fold. These were determined as adjusted Spots-Forming Cells (aSFCs) by cultured ELISPOT.

### Data processing and statistical analysis

To analyze and visualize the data, Microsoft Excel, GraphPad Prism 7, and FlowJo v10 (BD) were used. Statistical significance was determined by Wilcoxon test, Pearson’s correlation analysis, Welch’s t-test (with a two-tailed test of significance), one-way analysis of variance [ANOVA]. Values of P <0.05 were considered significant. The data are expressed as means ± standard deviation or standard error of the mean, and the sample sizes are presented in the figures.

## Results

### CD4^+^ T cell responses to *M. tuberculosis* antigens by *ex vivo* and cultured ELISOT

**Figure 1:**
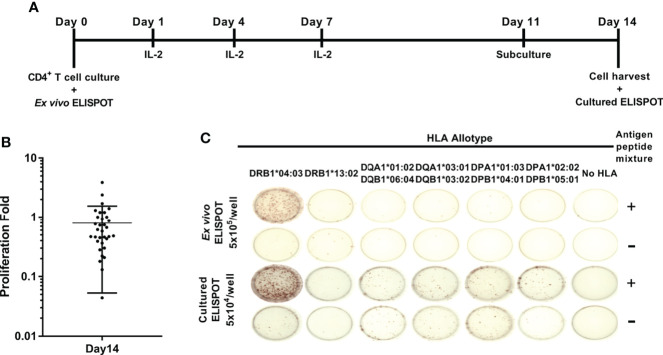
Schematic representation of *ex vivo* and cultured ELISPOT. **(A)** *In vitro* culture of antigen-specific CD4^+^ T cell for cultured ELISPOT. As the *M. tuberculosis* antigens, a mixture of peptide pools of TB10.4, Ag85b, ESAT-6, and CFP-10 was used. **(B)** The proliferation folds of CD4^+^ T cells during 14 days of culture. Error bars indicate the mean ± standard deviation. **(C)** Representative *ex vivo* ELISPOT and cultured ELISPOT of HD04 using aAPCs expressing a single HLA class II allotype pulsed without antigens **(-)** or with *M. tuberculosis* antigens (+). *Notation of the HLA allele.

Peptide pools of TB10.4, Ag85b, ESAT-6, and CFP-10 were used to assess CD4^+^ responses to *M. tuberculosis* according to a single HLA class II allotypes in healthy donors. HLA-DRB1, -DQA1, -DQB1, -DPA1, and -DPB1 alleles were genotyped in 33 healthy donors (**Table 1**). The single HLA class II allotype-expressing aAPCs were established for those whose alleles were typed. The CD4^+^ T cells from peripheral blood were stimulated by aAPCs pulsed with the *M. tuberculosis* antigens. *M. tuberculosis* antigen-specific CD4^+^ T cell responses were measured by IFN-γ ELISPOT assay and were designated to *ex vivo* ELISPOT (**Figure 1**). Cultured ELISPOT was conducted to measure the responses of memorial CD4 T cells by culturing with *M. tuberculosis* antigens for 14 days (**Figure 1A**). Individual donors showed various values of proliferation fold. The average of cell proliferation fold during the culture was 0.8 (min 0.04, max 3.8) (**Figure 1B**). Using aAPCs expressing a single HLA class II allotype, CD4^+^ T cell responses by HLA class II allotypes possessed by each donor were measured with *ex vivo* ELISPOT and cultured ELISPOT (**Figure 1C**). Cultured CD4^+^ T cells were analyzed expression of CCR7 and CD45RO to define T cell subsets (**Supplementary Figure S3**). The cultured CD4^+^ T cells were CD45RO^+^CCR7^-^ which indicates cultured CD4^+^ T cells are effector memory.

### CD 4^+^ T cell responses to *M. tuberculosis* antigens according to HLA class II loci

**Figure 2:**
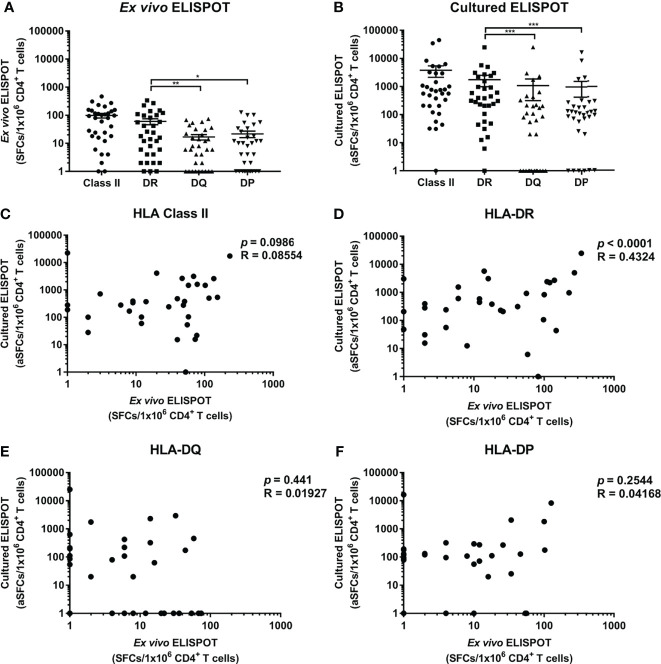
Frequencies of CD4^+^ T cells specific for *M. tuberculosis* antigens restricted by HLA class II loci and correlation between *ex vivo* and cultured ELISPOT. **(A)** *Ex vivo* ELISPOT, **(B)** Cultured ELISPOT. Statistical analysis was performed using Wilcoxon test. *p <0.05, **p < 0.01, ***p < 0.001. Error bars present mean ± standard error of the mean. The frequencies *M. tuberculosis*-specific CD4^+^ T cells were adjusted to the frequency per 1×10^6^ CD4^+^ cells in peripheral blood to compare *ex vivo* and cultured ELISPOT. Correlation between *ex vivo* and cultured ELISPOT of HLA class II **(C)** which is the sum of the responses by all HLA class II allotypes, and of each HLA-DR **(D)**, -DQ **(E)** and -DP **(F)** locus.

CD4^+^ T cell responses specific to *M. tuberculosis* antigens were analyzed by HLA-DR, -DQ, and -DP loci to compare the responses by each locus (**Figure 2**). The response by a locus was calculated by summing the responses by two alleles of a locus. The response by HLA-DR was higher than by HLA-DQ and -DP within donors in *ex vivo* ELISPOT (**Figure 2A**, Wilcoxon test, DR vs. DQ *p* = 0.0051; DR vs. DP *p* = 0.0136). There was no significant difference in responses by HLA-DQ and -DP (Wilcoxon test, *p* = 0.6939).

Next, the responses of memory T cells were measured by cultured ELISPOT. The response in cultured ELISPOT was increased 39-fold compared to *ex vivo* ELISPOT (3791 SFCs per a million in cultured ELISPOT; 98 SFCs per a million in *ex vivo* ELISPOT). In cultured ELISPOT, the response by HLA-DR was higher than by HLA-DQ and -DP, consistent with *ex vivo* ELISPOT (**Figure 2B**, Wilcoxon test, DR vs. DQ *p* = 0.0002; DR vs. DP *p* = 0.0002). The response by HLA-DQ was similar to the response by HLA-DP, as in *ex vivo* ELISPOT (Wilcoxon test, *p* = 0.7259). In the sum of the responses by loci, there was no pattern in the responses by HLA class II in cultured and *ex vivo* ELISPOT (**Figure 2C**, Pearson’s correlation, *p* = 0.0986). According to the analysis of the response by each locus, the response by HLA-DR was correlated in cultured and *ex vivo* ELISPOT (**Figure 2D**, Pearson’s correlation, *p* < 0.0001). On the contrary, the response by HLA-DQ and -DP was not correlated in cultured ELISPOT and *ex vivo* ELISPOT, and it was detected only in cultured ELISPOT or only in *ex vivo* ELISPOT (**Figure 2E** **, F**, HLA-DQ *p* = 0.441; HLA-DP *p* = 0.2544).

### CD4^+^ T cell responses according to a single HLA class II allotypes

**Figure 3:**
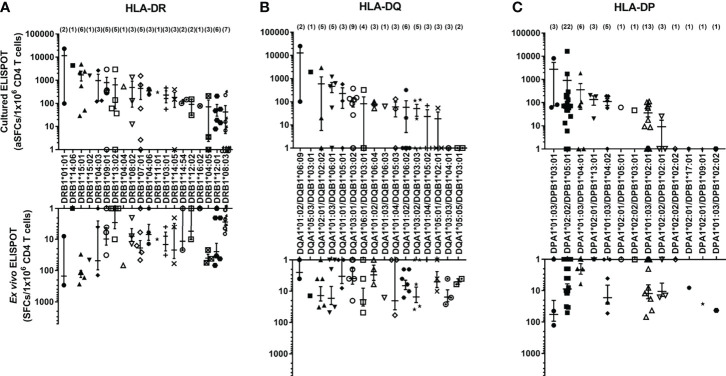
The frequencies of antigen-specific CD4*^+^* T cells by allotypes. The frequencies of *M. tuberculosis* antigen-specific CD4*^+^* T cells by HLA-DR **(A)**, -DQ **(B)**, and -DP **(C)** allotypes measured by *ex vivo* and cultured ELISPOT. The parentheses indicate the number of donors with the allotype. Error bars present mean ± standard error of the mean.

Using aAPCs expressing a single HLA class II allotype, the CD4^+^ T cell responses by HLA class II allotypes possessed by each individual were measured with *ex vivo* ELISPOT and cultured ELISPOT, and the distribution of responses to each allotype was investigated. In cultured ELISPOT, DRB1*01:01, DRB1*14:06, DRB1*15:01, DRB1*15:02, DRB1*04:03, DRB1*09:01, DRB1*13:02, DRB1*04:04, DRB1*08:02, DRB1*07:01, DRB1*04:06, DRB1*11:01 and DRB1*03:01 among 20 HLA-DR allotypes (**Figure 3A**), DQA1*01:02/DQB1*06:09, DQA1*05:03/DQB1*03:01, DQA1*02:01/DQB1*02:02, DQA1*01:03/DQB1*06:01 and DQA1*01:01/DQB1*05:01 among 16 HLA-DQ allotypes (**Figure 3B**), and, DPA1*01:03/DPB1*03:01, DPA1*02:02/DPB1*05:01 and DPA1*01:03/DPB1*04:01 among 13 HLA-DP allotypes (**Figure 3C**) showed an average of more than 200 aSFCs per a million and they showed a high response in the order listed. Most of allotypes in *ex vivo* ELISPOT showed less than 200 SFCs per a million.

### CD4^+^ T cell responses restricted by each HLA class II allotypes within individuals

**Figure 4:**
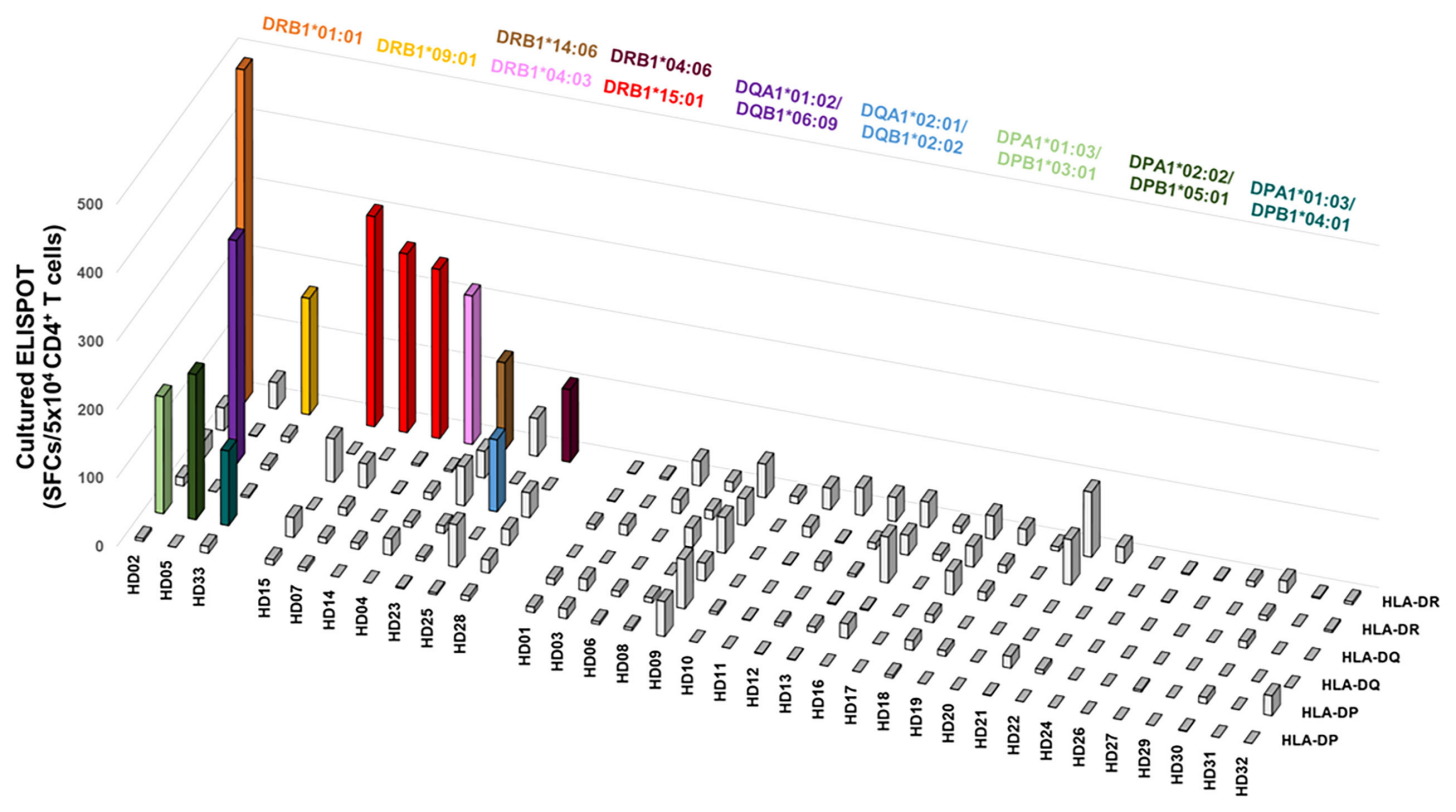
The distribution of CD4^+^ T cell responses by an HLA class II allotype within individuals. Colored bars indicate allotypes with positive responses higher than 100 SFCs per 5×10^4^ cells. Each color represents the corresponding allotype.

CD4^+^ T cell responses restricted by an HLA class II allotype by cultured ELISPOT were analyzed in individual donors to investigate the allotype dominance to *M. tuberculosis* antigens. A positive response was defined in the case that the response by an allotype is higher than 100 SFCs per 5×10^4^. Of the 33 donors, ten showed a positive response in an allotype; seven showed a positive response by only one allotype; three showed a positive response by two allotypes (**Figure 4**). The remaining 23 donors did not show a positive response in any allotype. We classified the response by an HLA allotype in the order of responses’ magnitude to compare the response without an arbitrary threshold in a donor. The highest response by an allotype was higher than the second, third, and fourth highest responses by the other allotypes (**Supplementary Figure S4**, one-way ANOVA, 1^st^ vs. 2^nd^ *p* = 0.0033, 1^st^ vs. 3^rd^ and 1^st^ vs. 4^th^ *p* < 0.0001). And we also classified the response by an HLA allotype of individuals showed positive responses in cultured ELISPOT in the order of responses’ magnitude. The highest response by an allotype was higher than the second, third, and fourth highest responses by the other allotypes (**Supplementary Figure S5**, one-way ANOVA, 1^st^ vs. 2^nd^ *p* = 0.0429, 1^st^ vs. 3^rd^ *p* = 0.0144, and 1^st^ vs. 4^th^ *p* = 0.0127). Allotype dominance exists in *ex vivo* ELISPOT within individuals having allotypes showed positive responses in cultured ELISPOT. These results suggest that there is an allotype dominance even in the *M. tuberculosis* antigens.

**Figure 5:**
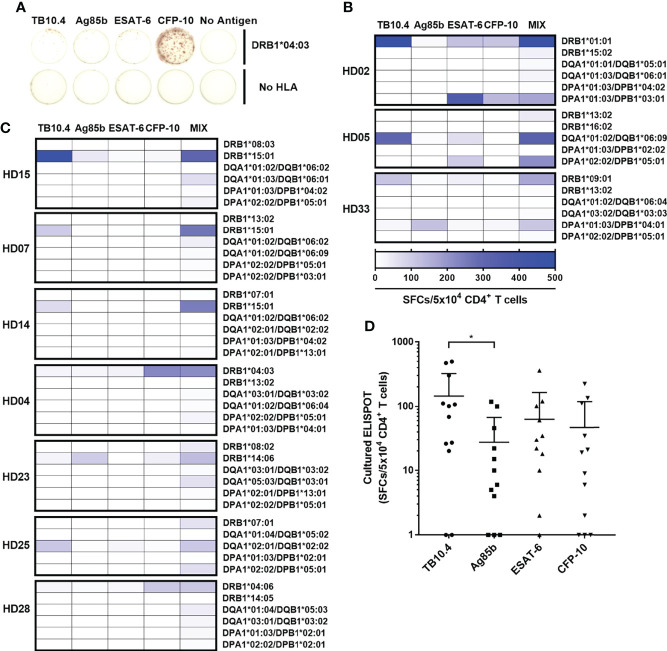
CD4^+^ T cell responses to each *M. tuberculosis* antigens TB10.4, Ag85b, ESAT-6, and CFP-10 by an HLA class II allotype. **(A)** Representative cultured ELISPOT to each antigen in HD04. CD4^+^ T cell responses by two allotypes **(B)** or an allotype **(C)** in a donor that showed a positive response were measured individually to TB10.4, Ag85b, ESAT-6, CFP-10, and a mixture of antigens. **(D)** Distribution of CD4^+^ T cell responses to each *M. tuberculosis* antigens. Statistical analysis was performed using Welch’s t-test. *p = 0.0373. Error bars present mean ± standard deviation.

The responses of CD4^+^ T cells to a single HLA class II allotypes have been assessed using a mixture of four *M. tuberculosis* antigens. We further defined which of the four *M. tuberculosis* antigens mainly used for the positive response by an allotype. (**Figure 5A**). In *ex vivo* ELISPOT, all HLA class II allotypes except 2 allotypes showed less than 100 SFCs per 5×10^5^, which was not suitable for subsequent analysis (**Supplementary Figure S6**). Even in the three donors who showed positive responses by two allotypes, the each allotype had responses predominantly to a single antigen (**Figure 5B**). The positive responses by two allotypes targeted to different proteins of *M. tuberculosis*. In HD02, DRB1*01:01 and DPA1*01:03/DPB1*03:01 showed antigen specificity for TB10.4 and for ESAT-6, respectively. In HD05, DQA1*01:02/DQB1*06:09 and DPA1*02:02/DPB1*05:01 showed antigen specificity for TB10.4 and for ESAT-6. In HD33, DRB1*09:01 and DPA1*01:03/DPB1*04:01 showed antigen specificity for Ag85b and for TB10.4.

The seven donors that showed a positive response by a single allotype showed a response to only one antigen (**Figure 5C**). The DRB1*15:01 showed an antigen specificity for TB10.4 in HD15, HD07 and HD14. The DRB1*15:01-restricted responses targeted for the same antigen, which is TB10.4. The DRB1*04:03 showed an antigen specificity for CFP-10 in HD04. The DRB1*14:06 showed an antigen specificity for Ag85b in HD23. The DQA1*02:01/DQB1*02:02 showed an antigen specificity for TB10.4 in HD25. The DRB1*04:06 showed an antigen specificity for CFP-10 in HD28. Comparing the immunogenicity of each antigen based on the positive responses by an allotype, the average frequencies of the CD4^+^ T cell responses to each antigen were 135 SFCs/5×10^4^ in TB10.4; 59 SFCs per 5×10^4^ in ESAT-6, 50 SFCs per 5×10^4^ in CFP-10; 26 SFCs per 5×10^4^ cells in Ag85b (**Figure 5D**). The CD4^+^ T cell responses were significantly higher to TB10.4 than Ag85b (Welch’s t-test. *p* = 0.0373). These results suggest that HLA class II allotype dominance targeted predominantly to a single *M. tuberculosis* antigen (**Supplementary Figure S7**).

## Discussion

The *ex vivo* ELISPOT for *M. tuberculosis* is mainly used for the diagnosis of infected patients. In this study, the cultured ELISPOT was used to measure the distribution of memory CD4^+^ T cells induced by past asymptomatic *M. tuberculosis* infection or vaccine-induced in healthy donors. These data suggest that it is necessary to measure the memory CD4^+^ T cell response by stimulation and *in vitro* culture because of the low T cell response to *M. tuberculosis* in healthy individuals (**Figures 2A, 9B**). The cultured ELISPOT measured the Hepatitis B virus-specific T cell response more sensitively than *ex vivo* ELISPOT in patients with chronic disease (34). For Dengue virus (DENV)-specific T cells, the cultured ELISPOT determined the silent and symptomatic transmission of DENV and identified the DENV-specific immune response for the assessment of severity (35, 36). Although the cultured ELISPOT can measure lower immune responses compared to *ex vivo* ELISPOT, cultured ELISPOT carries potential distortions associated expansion phase. In particular, HLA-DR-restricted CD4^+^ T cell responses showed a correlation between *ex vivo* and cultured ELISPOT results but not the HLA-DQ or DP (**Figures 2D** **–F**).

The CD4^+^ T cell response to *M. tuberculosis* antigens was higher in HLA-DR than HLA-DQ and HLA-DP in *ex vivo* ELISPOT (**Figure 2A**), similar to the CD4^+^ T cell response to CMV-pp65 and SARS-CoV-2 antigens (32, 37). The expression of HLA-DR was higher than HLA-DQ and -DP in PBMCs (32). It suggests that T cells restricted by HLA-DR are highly selected against pathogens. The genes encoding α chains show highly polymorphism only in HLA-DQ compared to that of HLA-DR and -DP. The high polymorphism of α chains in HLA -DQ may contribute to the low probability of dominant responses competing for the antigen presentation (38–40).

Although the mixture of four *M. tuberculosis* antigens was used, only one or two allotypes were used in CD4^+^ T cell response among six allotypes in an individual (**Figure 4**). The one allotype-restricted CD4^+^ T cells responded primarily to one antigen (**Figure 5C**). Even in the donors with the positive responses by two allotypes, each allotype showed a response to a different antigen (**Figure 5B**). These results suggest that allotype dominance exists despite the use of a mixture of multiple *M. tuberculosis* antigens. The T cell response to pp65 of CMV and spike, nucleocapsid, and membrane of SARS-Cov-2 also showed the allotype dominance within an individual (32, 33, 37). At early time points after infection, diverse high-affinity clones were stimulated: however, at late time points, a few low-affinity clones predominated in CMV-pp65-specific T cell responses (41, 42). The allotype dominance in *M. tuberculosis*-specific CD4^+^ T cells might be the survival of T cells by competing for affinity with bacterial peptides and HLA allotypes under infection.

Because the mRNA expression of HLA class II allotypes can be reduced by several factors such as 19-Kd lipoprotein of M. tuberculosis, it should be analyzed the mRNA level of selected HLA class II allotypes according to the different stages and CD4^+^ T cell responses depending on the expression of various mRNAs of the HLA class II allotypes in the future (43).

The distribution of CD4^+^ T cell responses to TB10.4 was significantly higher than Ag85b (**Figure 5D**). TB10.4 and CFP-10 were recognized most frequently by more than 40% of the donors among *M. tuberculosis* antigens used in TB vaccines or in IFN-γ release assay (44). The ESAT-6 was recognized by more than 20% of the donors, while Ag85b was recognized in less than 10% of donors. According to the Tuberculist database, TB10.4, ESAT-6, and CFP-10 antigens, which are highly immunogenic, are classified as the cell wall and cell processes category (45). Ag85b, which is less immunogenic, is involved in lipid metabolism.

The DRB1*08:03 and DQB1*06:01 alleles were associated with TB in previous studies (13, 46). DRB1*08:03 showed the lowest response among the HLA-DR alleles (**Figure 3A**). However, the combination of DQB1*06:01 and DQA1*01:03 had a high response (**Figure 3B**). The disease association studies focused on only an alpha chain or a beta chain of HLA-DQ alleles. Both alpha and beta chains of HLA-DQ are highly polymorphic and combine to form an allotype. Therefore, the association of TB and HLA should be investigated by the combination of alpha and beta chains rather than by an allele.

In this study, ten donors were positive as measuring CD4^+^ T cell responses to four M. tuberculosis antigen mixtures using cultured ELISPOT in 33 healthy donors. This frequency was similar to that of latent tuberculosis in the global population (1). The research should be preceded to distinguish between latent and active tuberculosis.

In summary, dominant allotypes to the *M. tuberculosis* antigens were identified by measuring memory T cell response restricted by an HLA class II allotype using cultured ELISPOT. Moreover, there was an allotype dominance that CD4^+^ T cell response appeared only by a single allotype in an individual. The CD4^+^ T cells restricted by an allotype responded to one antigen. These results will provide valuable information to investigate the distribution of past or asymptomatic infections in the region and can also be used to rationally determine the type and number of antigens included in the vaccine by estimating the proportion of people who respond to the vaccine in a specific population group with a different HLA class II distribution. The dominant allotype-restricted epitopes should be elucidated, though epitopes of *M. tuberculosis* antigens were reported. There are two genes encoding the alpha and beta chain in HLA-DQ and HLA-DP, so there is a total of four combinations. However, only two allotypes of a haplotype were used for the analysis in this study. The responses by four combinations of HLA-DQ and -DP should be investigated in future research.

## Data Availability Statement

The datasets presented in this study can be found in online repositories. The names of the repository/repositories and accession number(s) can be found below:

https://www.ncbi.nlm.nih.gov/bioproject/PRJNA721949/.

## Ethics Statement

The studies involving human participants were reviewed and approved by Institutional Review Board of the Catholic University of Korea (MC21SESI0005). The patients/participants provided their written informed consent to participate in this study.

## Author Contributions

Y-HL, Y-SH, and T-GK conceived and designed the experiments. Y-HL, Y-SH, and H-AJ performed experiments. S-MK and H-JS contributed reagents/materials/analysis tools. I-CB typed the HLAs. Y-HL, Y-SH, and T-GK analyzed data and wrote the manuscript. All authors contributed to the article and approved the submitted version.

## Funding

This study was supported by a grant of the Korean Health Technology R&D Project, Ministry for Health &Welfare, Korea (HI14C3417).

## Acknowledgments

We thank the Catholic Hematopoietic Stem Cell Bank, College of Medicine, The Catholic University of Korea, Seoul, South Korea, for typing HLA.

## Conflict of Interest

The authors declare that the research was conducted in the absence of any commercial or financial relationships that could be construed as a potential conflict of interest.

## Publisher’s Note

All claims expressed in this article are solely those of the authors and do not necessarily represent those of their affiliated organizations, or those of the publisher, the editors and the reviewers. Any product that may be evaluated in this article, or claim that may be made by its manufacturer, is not guaranteed or endorsed by the publisher.
