# Supplementary material for: Comprehensive analysis of mycobacterium tuberculosis antigen-specific CD4+ T cell responses restricted by single HLA class II allotype in an individual
Source: Front Immunol. 2022 Jul 28;13:897781. doi: 10.3389/fimmu.2022.897781 (PMC9366214; doi:10.3389/fimmu.2022.897781)
Supplement: Supplementary file 1 [file DataSheet_1.docx]

Supplementary Material

# Supplementary Figures

**
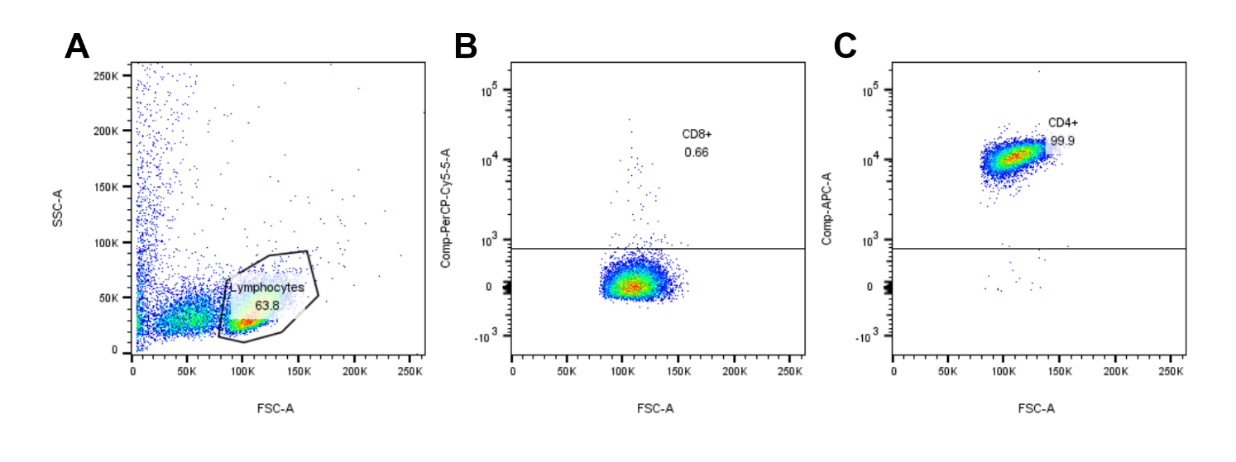
**

**Supplementary Figure 1. Flow cytometry analysis of isolated CD4^+^ T cells.** **(A)** Lymphocytes gating, **(B)** proportion of CD8^+^ T cells and **(C)** CD4^+^ T cells in isolated CD4^+^ T cells.


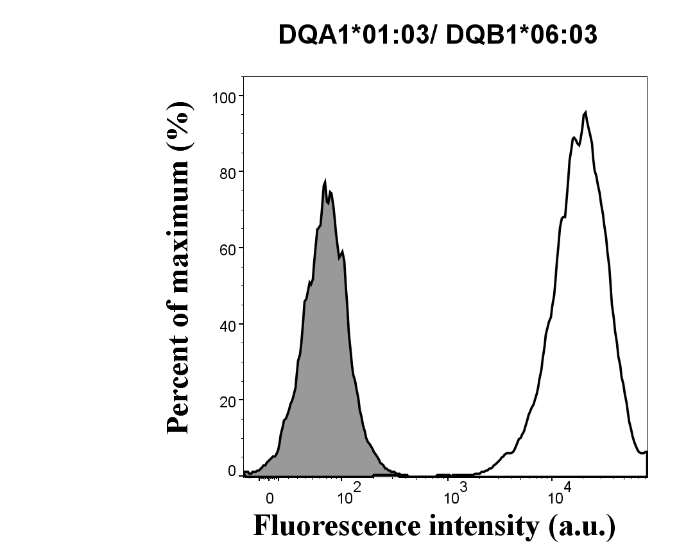


**Supplementary Figure 2.** Expression of HLA-DQA1*01:03/DQB1*06:03 allotype on aAPC.

**
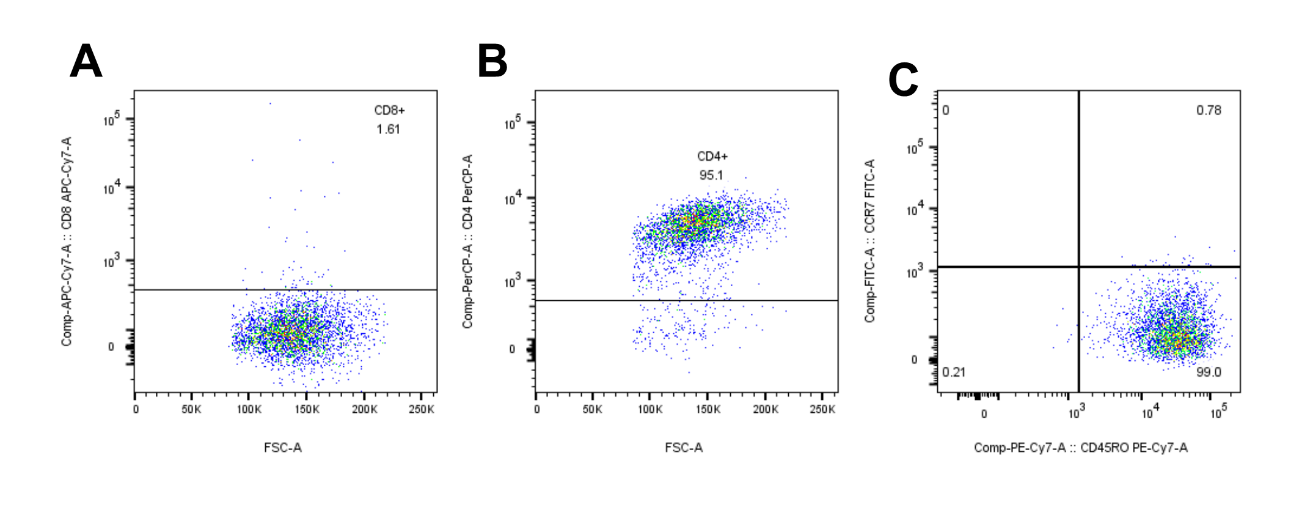
**

**Supplementary Figure 3. Flow cytometry analysis of cultured CD4^+^ T cells**. **(A)** Proportion of CD8^+^ T cells and **(B)** CD4^+^ T cells in cultured CD4^+^ T cells. **(C)** Surface expression of CCR7 and CD45RO.

**
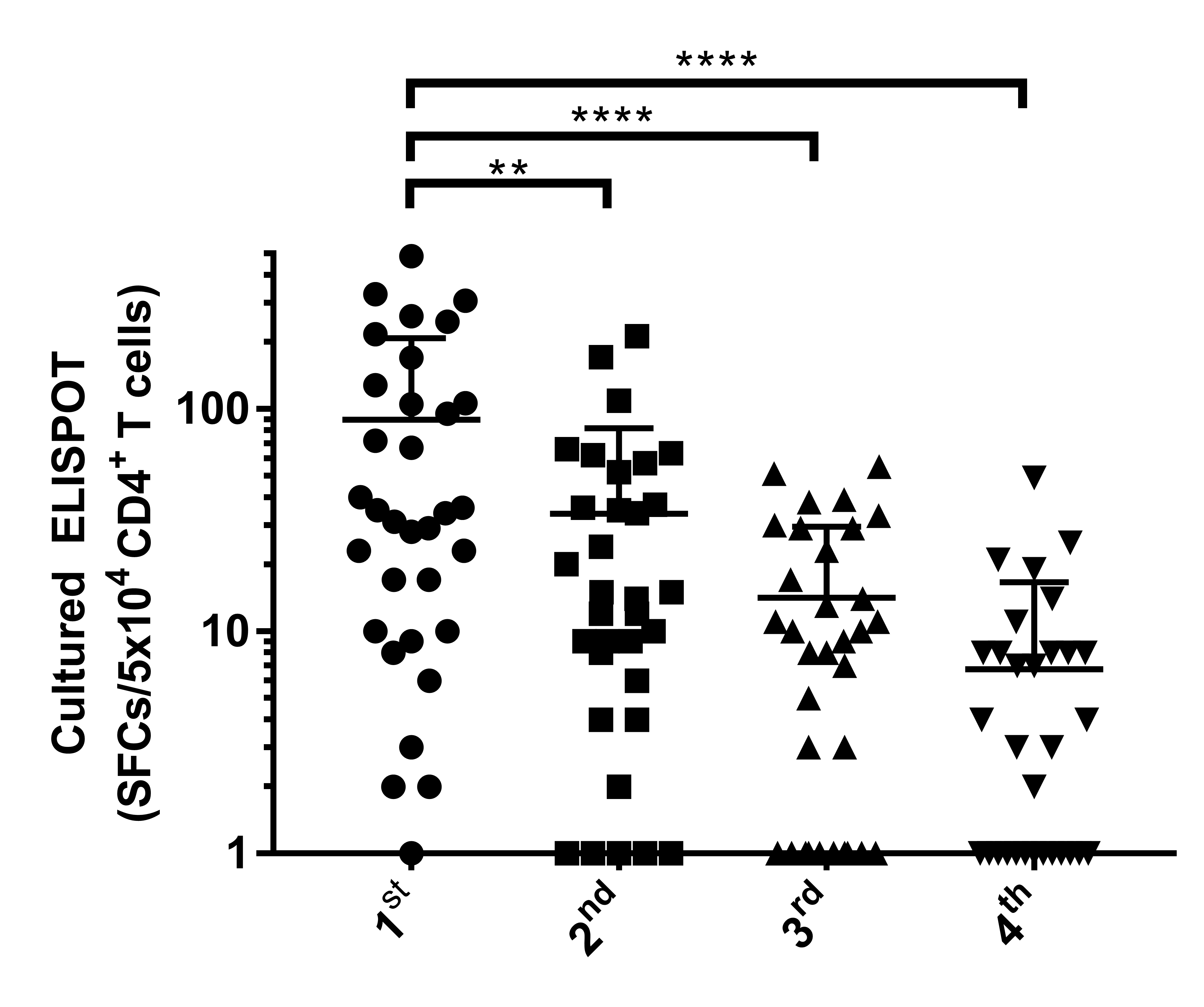
**

**Supplementary Figure 4.** The responses within individuals were analyzed by the order of highest response by alleles of an individual. Error bars present mean ± SD. Statistical analysis was performed using one-way ANOVA. ***p* = 0.0033, *****p* < 0.0001.





**Supplementary Figure 5.** The responses within individuals showed positive responses in cultured ELISPOT were analyzed by the order of highest response (n=10). Error bars present mean ± SD. Statistical analysis was performed using one-way ANOVA. **p* < 0.05.

**
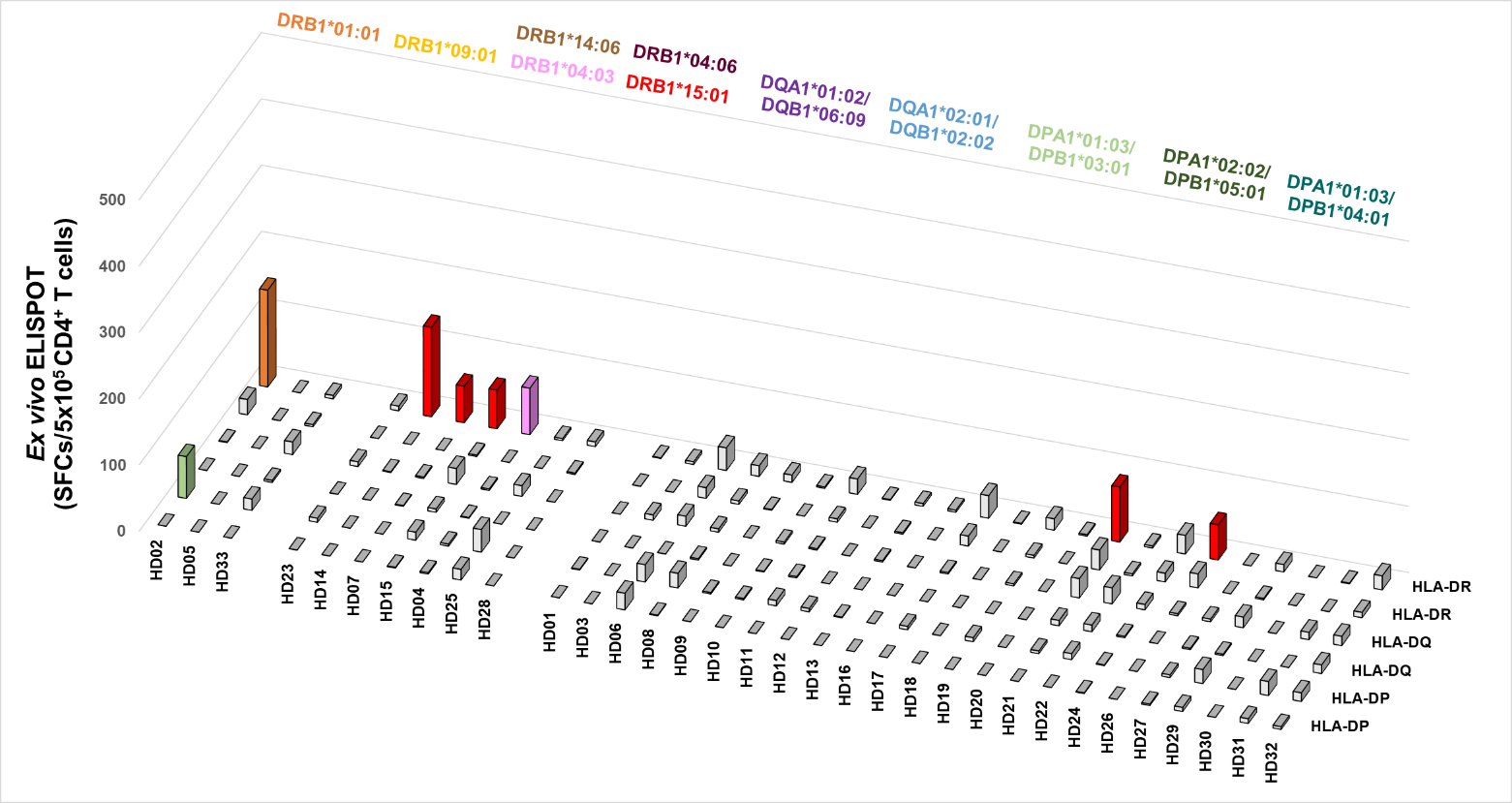
 Supplementary Figure 6**. Distribution of CD4^+^ T cell responses specific for *M. tuberculosis* antigens to HLA class II allotypes within individuals (n=33). Colored bars indicate allotypes with positive responses higher than 50 SFCs per 5×10^5^ cells. Each color represents the corresponding allotype.


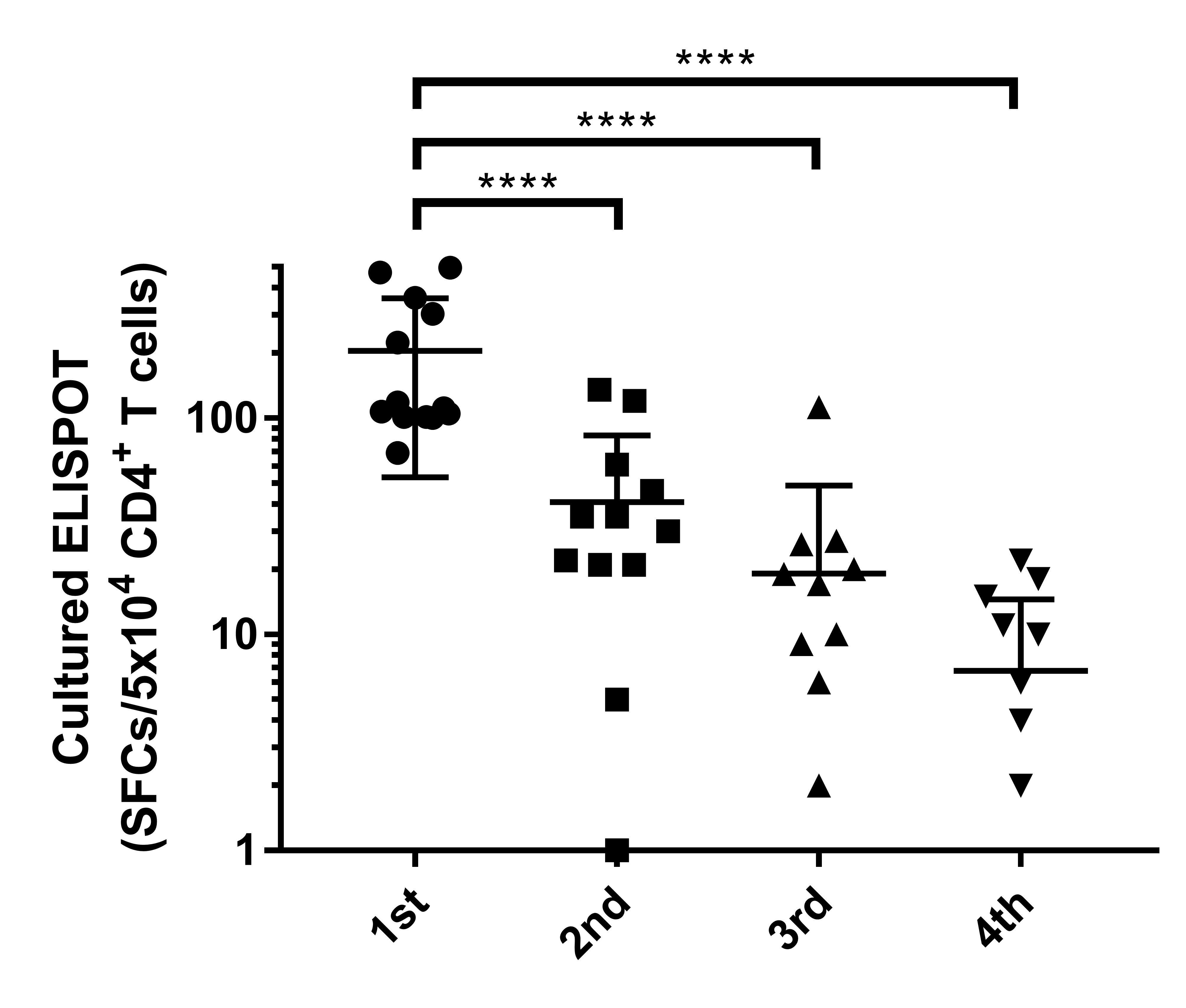


**Supplementary Figure 7.** The responses of CD4^+^ T cells were analyzed by the order of highest response by antigen. Error bars present mean ± SD. Statistical analysis was performed using one-way ANOVA. *****p* < 0.0001.
